# Abdominal wall pseudocyst as a complication of ventriculoperitoneal shunt insertion: a case report

**DOI:** 10.11604/pamj.2022.41.23.29426

**Published:** 2022-01-10

**Authors:** Marsal Risfandi, Celia Celia, Robert Shen

**Affiliations:** 1 Department of Neurosurgery, Faculty of Medicine, University of North Sumatra, Sumatera Utara, Indonesia,; 2 Department of Neurosurgery, Putri Bidadari General Hospital, Jalan Stabat Tanjung Pura Langkat, Sumatera Utara, Indonesia,; 3 Department of Surgery, School of Medicine and Health Sciences, Atma Jaya Catholic University of Indonesia, Jalan Pluit Raya, Jakarta, Indonesia,; 4 Atma Jaya Neuroscience Research (ANR), Master Study Program in Biomedical Sciences, School of Medicine and Health Sciences, Atma Jaya Catholic University of Indonesia, Jalan Pluit Raya, Jakarta, Indonesia

**Keywords:** Abdominal wall, pseudocyst, hydrocephalus, diagnosis, case report

## Abstract

The abdominal cavity has long been used to absorb cerebrospinal fluid (CSF) in a ventriculoperitoneal (VP) procedure in hydrocephalus patients. Although this procedure is quite common, some complications can potentially arise. This study aimed to report a case of pseudocyst formation as a rare complication (<5% incidences) following a VP shunt insertion. A case of a 62-year-old male patient with a history of communicating hydrocephalus post-VP shunt insertion presented with symptoms of acute progressive right abdominal pain and was found a formation of large abdominal wall pseudocyst. An upper abdominal computed tomography (CT) scan showed a well-defined cystic mass lesion located intraperitoneally in the right hypochondrium. He subsequently underwent an exploratory laparotomy and surgical excision of the pseudocyst, followed by improved symptoms experienced. Clinicians must be aware of this complication because early diagnosis and prompt management will eventually improve outcomes for reducing abdominal pain and improving the patient's quality of life.

## Introduction

Ventriculoperitoneal (VP) shunt placement is a common neurosurgical procedure performed to manage hydrocephalus. Although complications from this procedure are rare, VP shunt still can cause complications, including abdominal wall perforation, peritonitis, and ascites [1]. Abdominal wall pseudocyst formation is a rare occurrence but one of the well-explained complications resulting from the VP shunt procedure [2-4]. Herein, we presented an unusual case of a patient with right abdominal pain at the right hypochondrium region and was subsequently, after the examination procedure, found to have a massive CSF pseudocyst attributed to VP shunt malfunction.

## Patient and observation

**Patient information:** a 62-year-old male patient, Caucasian, was admitted to our hospital with the chief complaint of acute progressive right abdominal pain on the day of admission; the pain showed locally 3cm below the right hypochondrium region. The patient had a history of communicating hydrocephalus post-VP shunt insertion. Prior to the complaint, there was no history of abdominal trauma or identifiable toxic and spicy food consumption. Never had a similar complaint based on patient history or family history, and had no history of gastrointestinal related disease. The history of the patient's genetic information is unknown. The patient has an overweight body mass index and sedentary lifestyle, is non-alcoholic, and a non-smoker.

**Clinical findings:** on examination, the general appearance of the patient was lethargic, in an obvious discomfort and moderate to severe pain according to visual analogue scale (VAS), vital signs were stable within normal limits. On abdominal examination, there was no abdominal distension, but occurred to be rebound tenderness in the right upper abdominal region and decreased of the bowel sounds.

**Timeline of current episode:** on January 2021, whole events occur in order sequences: hospital admission, anamnesis and data collection, physical examination, laboratory testing (complete blood count and liver function), radiologic examination using computed tomography (CT) scan, generating working diagnosis, patient education, therapeutic interventions and follow-up.

**Figure 1:**
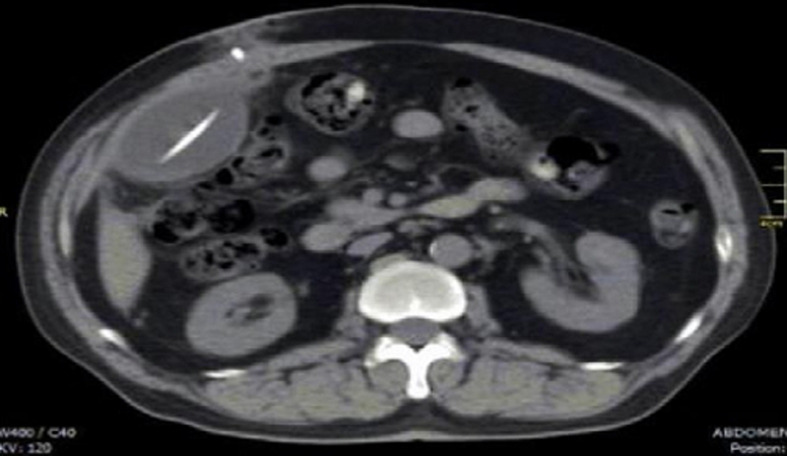
axial CT showing abdominal fluid collection adjacent to ventriculoperitoneal shunt catheter tip located on right abdomen

**Diagnostic assessment:** the patient's conditions are unlikely acute appendicitis based on the Alvarado scoring system (score 1), with only rebound tenderness, no pain migration to the right iliac fossa, no anorexia, no nausea and vomiting, no right iliac fossa (RIF) tenderness, no fever, and a complete blood count (CBC) happened to be normal. There was also no hepatomegaly with normal liver function by laboratory testing, and no costovertebral angle (CVA) tenderness indicating a normal condition of liver and renal. Radiologic findings using upper abdominal computed tomography (CT) scan showed a collection of homogeneous low-density fluid, measuring 10cm × 8cm × 7cm, adjacent to the catheter tip of the VP shunt (Figure 1). After reviewing the imaging studies, including a CT scan of the upper abdomen, it appears that the cyst was caused by loculations and septations around the end of the peritoneal VP shunt catheter. There were no challenges in access to diagnostic testing, financial or cultural issues, and medical assessment to intervention.

**Diagnosis:** intraperitoneal CSF cysts and abdominal wall pseudocyst et causa complication of VP shunt insertion as the final diagnosis has been made.

**Figure 2:**
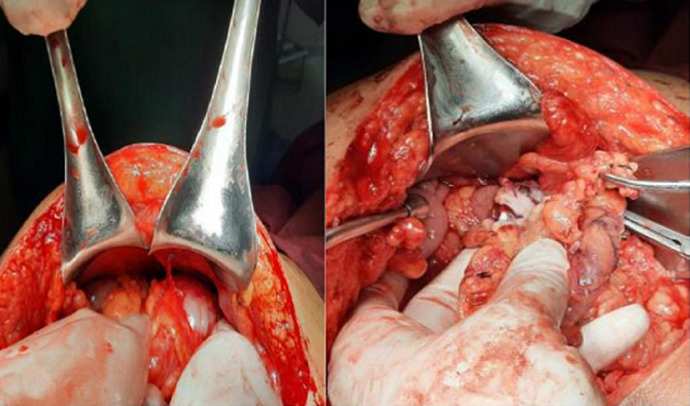
intraperitoneal pseudocyst and 750ml of clear fluid were drained, left picture, pseudocyst before aspiration; right picture, pseudocyst after aspiration, the distal shunt was identified

**Figure 3:**
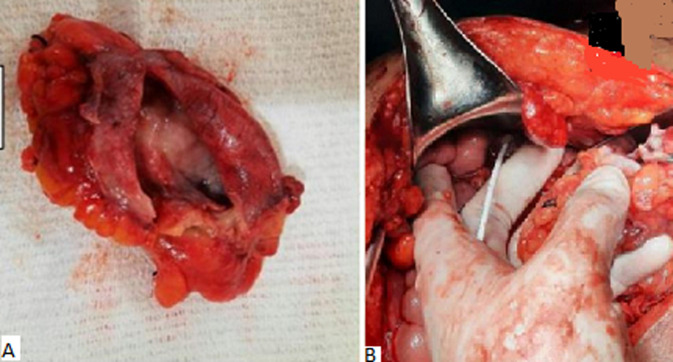
A) intraperitoneal cyst 10cm x 8cm x 7cm was excised; B) the distal side of the peritoneal shunt catheter was reinserted to the abdominal cavity

**Therapeutic interventions:** the patient was then admitted to the operating room for an exploratory laparotomy of intraperitoneal CSF cysts. Subsequently, the cystic mass was punctured through the abdominal wall, and 750ml of clear fluid was drained (Figure 2). The procedure was followed by surgical excision of the pseudocyst during the laparotomy. The distal side of the peritoneal shunt catheter was re-inserted to his abdominal cavity (Figure 3). Prophylactic ceftriaxone sodium 4g/day was administered for five days postoperatively.

**Follow-up and outcome of interventions:** the patient recovered very well after surgery, and there have been no additional complications following the procedure. The patient had no pseudocyst formation recurrences or similar complaints in the follow-up and was in good condition.

**Patient perspective:** “It was a very miserable and severe abdominal pain I have had in my whole life. Praise God and thank all the medical team, I feel relieved after surgery; the pain is gone, and only minimal pain in the post-surgery area in my abdomen. I just knew that the VP shunt procedure because of my hydrocephalus has potential complications. Hopefully, it won't happen again in the future.”

**Informed consent:** all informed consent for medical procedures and patient's medical information study was obtained orally and in writing recorded in the patient's medical record book at Putri Bidadari Hospital, Langkat, Indonesia. All ethical principles for medical research studies established by Putri Bidadari Hospital have been followed, and the ethical clearance committee approved this study.

## Discussion

Ventriculoperitoneal shunt insertion is a procedure performed to drain excessive CSF in which otherwise can lead to serious complications, including increased intracranial pressure and hydrocephalus formation. The risk of a VP shunt-related complication has been cited for varying from 1 to 59% throughout the literature [5-7]. Pseudocyst formation due to shunt insertion is mainly seen in the abdomen because most ventricular drains are placed in the peritoneal cavity, with a reported incidence of <5% [2,4,8]. Inadequate absorption, blockages, and abdominal adhesions are some of the common complications leading to the accumulation of CSF at the end of the catheter with subsequent pseudocyst formation [9]. Abdominal wall pseudocyst is defined as an accumulation of CSF at the distal end tip of the VP shunt within the abdominal cavity, or if the VP shunt has migrated, CSF accumulation occurs within the adjacent abdominal wall [1]. The term of “pseudocyst” is used referred as it is encapsulated by a fibrous, peritoneal membrane, which does not contain an epithelium [10]. Abdominal wall pseudocysts occur more commonly in children than adults and typically develop within five years of shunt placement or revision [11]. Pseudocysts present as a localized abdominal mass. Those with pseudocysts present with abdominal discomfort and diffuse abdominal tenderness. Depending on the pseudocyst's size, bowel obstruction symptoms may arise, including nausea and vomiting [12]. Abdominal CT scan remains the most common diagnostic modality, although ultrasonography (USG) has also been used in some cases. Computed tomography scan is often more helpful in differentiating those with severe abdominal pain as it can help identify other etiologies, such as appendicitis, diverticulitis, abdominal abscess, or bowel obstruction.

Pseudocysts can be identified on an abdominal CT scan as an extensive fluid-filled collection delimited by a thin wall adjacent to the catheter end tip (Figure 1). The differential diagnosis of abdominal cystic mass includes pancreatic pseudocyst, lymphocele, cystic lymphangioma, mesenteric cyst, benign cystic teratoma, seroma, cystic spindle-cell tumor, and cystic mesothelioma [13]. In this case, the absence of any infectious symptoms or history of the pancreatic disease combined with a history of VP shunt insertion in our patient significantly narrowed the differential diagnosis. Radiological findings and appearance during exploratory laparotomy also suggest a definite diagnosis of abdominal wall pseudocyst as a complication of VP shunt insertion. The exact pathogenesis of abdominal pseudocyst remains unknown; however, three different mechanisms have been proposed: chronic infection, foreign body reaction, and particle-like protein in CSF [14]. The onset for an abdominal pseudocyst to develop from the last shunting procedure ranged from three weeks to five years, with the longest reported cases in literature being eight years and fifteen years after VP shunt insertion [15,16]. The question of optimal treatment of abdominal pseudocysts has not been thoroughly answered. Treatment highly depends on the etiology, patient presentation, and clinical manifestations. Currently, several treatment methods have been described, including CT-guided fluid aspiration, paracentesis, and laparotomy with excision of cystic walls. In this case, our patient underwent exploratory laparotomy followed by cyst excision with re-insertion of the distal shunt catheter. The patient has a good clinical outcome and prognosis after the procedure with no recurrence during the follow-up assessment.

## Conclusion

Abdominal wall pseudocyst remains a rare but important complication of VP shunt insertion and is likely attributed to a low-grade inflammatory process. Clinicians other than neurosurgeons and pediatricians should be aware of this complication and keep it on the differential diagnosis for those who have a history of VP shunt insertion presenting with acute abdomen symptoms. Early recognition and intervention have been shown to improve clinical outcomes and limit unnecessary and costly diagnostic workups.
